# An analytical calculation of the fluid load support fraction in a biphasic material: An alternative technique

**DOI:** 10.1016/j.jmbbm.2012.04.015

**Published:** 2012-10

**Authors:** A.J.F. Stops, R.K. Wilcox, Z. Jin

**Affiliations:** Institute of Medical and Biological Engineering, Department of Mechanical Engineering, University of Leeds, Leeds, LS2 9JT, UK

**Keywords:** Fluid load support fraction, Cartilage, Poroelasticity

## Abstract

**Background:**

The fluid load support fraction (*W*^*F*^/*W*^*T*^) can be used to define the mechanical contribution of the interstitial fluid (*W*^*F*^) to the total force (*W*^*T*^) in the deformation of cartilage. Traditionally, *W*^*F*^/*W*^*T*^ is calculated using complex experimental setups or time-consuming micromechanical poroelastic Finite Element (FE) simulations.

**Aim:**

To define and validate a fast and efficient technique to predict *W*^*F*^/*W*^*T*^ using an analytical approach that can be applied without micromechanical detail or experimental measurement.

**Methodology:**

Poroelastic FE simulations defined accurate values of *W*^*F*^/*W*^*T*^ for a range of loading configurations and were used to validate subsequent predictions. The analytical prediction of *W*^*F*^/*W*^*T*^ used elastic contact mechanics to calculate *W*^*F*^, and viscoelastic FE representation to calculate *W*^*T*^. Subsequently, these independent calculations of *W*^*F*^ and *W*^*T*^ provided values of *W*^*F*^/*W*^*T*^ that were compared with the poroelastic FE calculations.

**Results and discussion:**

The analytical prediction of *W*^*F*^/*W*^*T*^ proved effective and suitably accurate (mean difference *S*<0.05). This technique demonstrated how *W*^*F*^ and *W*^*T*^ can be determined independently, without a biphasic constitutive model. Here we used viscoelasticity to calculate *W*^*T*^ as an example, however, *W*^*T*^ could be measured experimentally or predicted computationally.

## 1 Introduction

Cartilage is a biphasic material with two constitutive components that support load, a solid phase and a fluid phase. The fluid load support fraction (*W*^*F*^/*W*^*T*^) describes the fraction of total load (*W*^*T*^) that is supported by the interstitial fluid pore pressure (*W*^*F*^) [Ateshian et al., (2003); Ateshian (2009)]. Specifically, *W*^*F*^ is determined from the integration of the interstitial fluid pore pressure *p* over the contact area *A*,

(1) $$W^{F}=-\int_{A}pdA$$

and *W*^*T*^ from the integration of the contact pressure, where *n* is defined as the unit outward normal on *dA* of the stress *σ*, over the contact area *A*,

(2) $$W^{T}=-\int_{A}n\sigma ndA$$

Traditionally, experiments or micromechanical numerical analyses are used to quantify *W*^*F*^/*W*^*T*^, i.e. a counter-sunk pressure sensor has been used in experimental studies [Basalo et al., (2004); Krishnan et al., (2004); Park et al., (2003)], whereas micromechanical numerical studies [Soltz and Ateshian (1998)] have used poroelastic finite element (FE) simulations [Park et al., (2003); Pawaskar et al.*,* 2010, 2011]. Yet, as experimental studies generally measure isolated cartilage specimens, these techniques cannot measure *W*^*F*^/*W*^*T*^ during in-vitro physiological loads in whole-joints. Furthermore, given that poroelastic FE simulations are typically based on a quasi-static constitutive foundation, using an implicit solution method, these numerical calculations cannot model the dynamic conditions present in physiological loads [Stops et al., (2011)]. Thus, there is no constitutive framework that enables *W*^*F*^/*W*^*T*^ to be calculated in cartilage during dynamic analyses.

In light of the above, and considering the need to calculate *W*^*F*^/*W*^*T*^ in (1) a dynamic load condition and (2) in a whole joint: an explicit formulation of FE enables both a dynamic framework for these conditions. Though the quasi-static framework that underpins poroelasticity is not compatible with explicit FE, due to the problems in implementing inertial effects, explicit FE can model time-dependent effects using a phenomenological approach, e.g. viscoelasticity. The implementation of viscoelasticity does not allow for the micromechanical features of cartilage to be analysed, as poroelasticity allows, but it does provide a reliable representation of the macroscopic mechanics. Furthermore, a viscoelastic representation offers versatility in terms of modelling options, i.e. it can be used in a FE solution, and also a multibody dynamics solution (which is the primary technique for musculoskeletal modelling). Thus, in whole-joint analyses and in physiological loading conditions, viscoelasticity offers an ideal method to represent the time-dependent effects of cartilage.

Hence, a computational methodology that can predict *W*^*F*^/*W*^*T*^ alongside a viscoelastic representation would provide the opportunity to understand cartilage mechanics in a range of settings, from whole-joint analyses to dynamic loading conditions. Consequently, this paper aims to validate an analytical prediction of *W*^*F*^/*W*^*T*^ which complements a viscoelastic representation of cartilage, without the need for poroelastic simulations or complex experiments.

## 2 Methodology

### 2.1 Poroelastic *W*^*F*^/*W*^*T*^ calculation

**Fig. 1:**
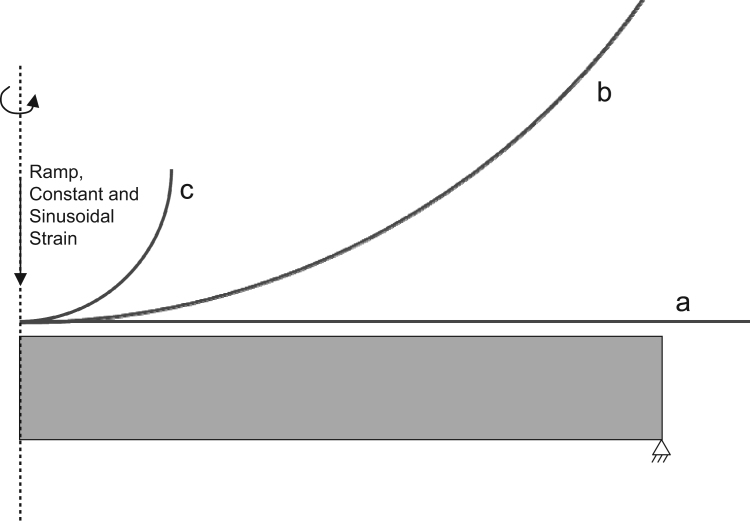
An illustration of the load setup for (a) a planar-surface indenter, (b) a large spherical indenter and (c) a small spherical indenter; note the indenter dimensions are for illustrative purposes only, please refer to Table 1 for accurate dimensions. The dimensions of the FE meshes were 12×2 mm, wherein the element size of each mesh (for each loading configuration) was optimised through a sensitivity analysis.

Poroelastic FE simulations were employed to calculate ‘gold standard’ values of *W*^*F*^/*W*^*T*^; such an approach has been shown to be accurate [Pawaskar et al.*,*, (2010, 2011)]. Axisymmetric models with contact-dependent fluid flow at the contact interface, free flow at the edges, and restricted flow at the bone–cartilage interface were generated. Uniaxial loads were applied through an indenter, perpendicular to the unconfined material, which was bonded to a rigid foundation (Table 1 and Fig. 1). Nine poroelastic FE simulations were performed: three indenter geometries included (1) a rigid planar-surface, (2) a large rigid sphere and (3) a small rigid sphere, all with (a) ramp loads, (b) constant strains (stress relaxation) and (c) sinusoidal strains (Table 1). In all cases, the mesh size was adjusted until convergence (Table 1) was achieved. For these nine conditions, *W*^*F*^/*W*^*T*^ was calculated based on Eqs. (1) and (2).

### 2.2 Analytical *W*^*F*^/*W*^*T*^ prediction

Using the *W*^*F*^/*W*^*T*^ values from the poroelastic simulations as a ‘gold standard’, in much the same way as experimental data is used to validate a computational simulation, the subsequent analytical predictions were compared. These analytical predictions were based on the concept that the two constituent phases of cartilage, the interstitial fluid and the deformable solid matrix, can be determined independently and that the mechanical contribution of the fluid can be approximated from the difference between the load supported by the solid matrix (*W*^*S*^) and the total load supported by the material (*W*^*T*^), $W^{F}=W^{T}-W^{S}$ [Bonnevie et al., (2011)]. Assuming the deformable solid matrix to be elastic, the value of *W*^*S*^ was calculated using elastic contact mechanics.

Here, three different equations were used to represent the different indenter geometries, as outlined below. In each case *E*_∞_ is the equilibrium elastic modulus, *h* is the thickness of the cartilage, *v*_∞_ is the equilibrium Poisson's ratio. The penetration depth *δ* and the total load *W*^*T*^ were determined from the respective viscoelastic simulation.

For the planar-surface configuration, *W*^*F*^/*W*^*T*^ was analytically predicted from the equation developed by Johnson (1985):

(3) $$\frac{W^{F}}{W^{T}}=1-\frac{W^{S}}{W^{T}}\;where\;W^{S}=\frac{(1-v_{\infty })E_{\infty }A_{1}\delta }{(1+v_{\infty })(1-2v_{\infty })h}$$

where $A_{1}=\pi a_{1}^{2}$ with *a*_1_ being the contact radius. Note, Eq. (3) is used to calculate the force in the solid phase of the material, and hence, all properties relate to the equilibrium state.

For the large spherical indenter, where the contact radius is large relative to the cartilage thickness, *W*^*F*^/*W*^*T*^ was calculated from:

(4) $$\frac{W^{F}}{W^{T}}=1-\frac{W^{S}}{W^{T}}\;where\;W^{S}=\frac{1-v_{\infty }}{\left(1+v_{\infty })(1-2v_{\infty }\right)}\frac{\pi E_{\infty }{a_{2}}^{4}}{4Rh}$$

where *R* is the radius of the sphere, *a*_2_ is the contact radius calculated by $\sqrt{2R\delta }$ [Jaffar (1997)].

And finally, for the small spherical indenter, where the contact radius is small relative to the cartilage thickness, *W*^*F*^/*W*^*T*^ was calculated from:

(5) $$\frac{W^{F}}{W^{T}}=1-\frac{W^{S}}{W^{T}}\;where\;W^{S}=\frac{4\pi E_{\infty }{a_{3}}^{3}}{(1-v_{\infty }^{2})RD}$$

where *D* is calculated as $D=3\pi +8\frac{a_{3}}{h}\left[-0.545{\left(\frac{a_{3}}{h}\right)}^{2}+\frac{1.768}{5}{\left(\frac{a_{3}}{h}\right)}^{4}\right]$ and *a*_3_ is the contact radius calculated by $\sqrt{R\delta }$; wherein a Poisson's ratio of 0.15 was assumed [Jaffar (2008)]. It should be noted that alternative asymptotic solutions to Eq. (5) also exist [e.g. Argatov, (2010); Argatov, (2011)].

In Eqs. (3–5), values of *W*^*T*^ were calculated from viscoelastic FE models whose properties (Table 1) were calibrated to the force–deformation response of the corresponding poroelastic model in the stress-relaxation setup. Both the instantaneous and the equilibrium response of the viscoelastic models proved accurate in representing the equivalent poroelastic models, as can be seen in Table 1 (Viscoelastic Calibration Error). Note, the mesh/configuration used for each loading scenario was identical in the respective poroelastic and viscoelastic models to ensure that there were no compounding influences on the solutions.

### 2.3 Statistical comparison

The nine analytical *W*^*F*^/*W*^*T*^ predictions were compared to the ‘gold standards’ using the mean difference (error) *S*,

(7) $$S=\frac{1}{n}\sum_{i=1}^{n}\sqrt{{\left(y_{i}^{G}-y_{i}^{A}\right)}^{2}}$$

where $y_{i}^{G}$ and $y_{i}^{A}$ are the *i*th increment from the ‘gold standard’ calculations and the analytical, respectively, while graphical illustrations are presented in terms of $\sqrt{{\left(y_{i}^{G}-y_{i}^{A}\right)}^{2}}$.

## 3 Results

### 3.1 Planar-surface indenter: *W*^*F*^/*W*^*T*^

**Fig. 2:**
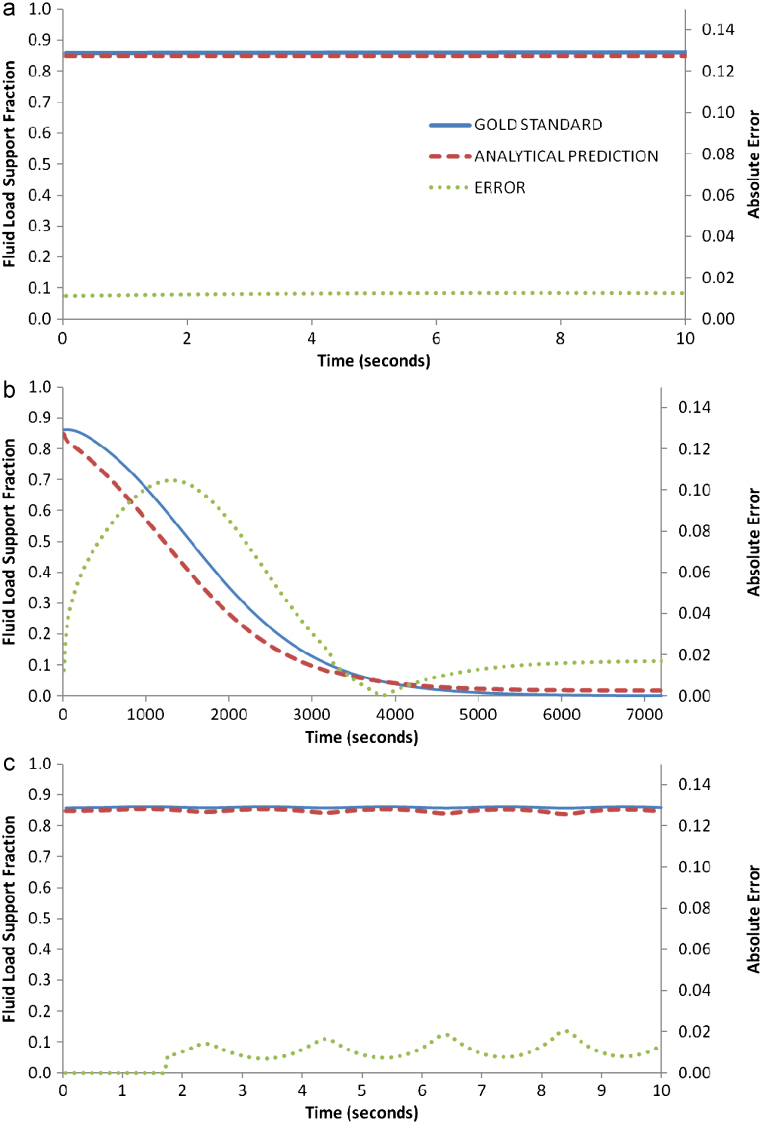
Values of the fluid load support fraction (*W*^*F*^/*W*^*T*^) calculated by a poroelastic FE simulation “gold standard” and predicted by an analytical calculation of the elastic contact mechanics (shown with errors defined as $\sqrt{{\left(y_{i}^{G}-y_{i}^{A}\right)}^{2}}$) during loads with a planar surface: (a) shows a linear ramp load up to 2.5% compressive strain (equivalent to 325 MPa load), (b) shows a constant 2.5% strain and, (c) shows a sinusoidal strain varying from 0.5 to 2.5% strain.

For the ramp load, the analytical *W*^*F*^/*W*^*T*^ prediction proved accurate, as can be defined by *S*=0.01 (Fig. 2a). Likewise, for the constant 2.5% strain, the predicted values were close to the ‘gold standard’, *S*=0.04 (Fig. 2b), and for the sinusoidal strain, the predicted values again followed the trend of the standard values, with *S*=0.07, but with discrepancies when the sine curve prescribed a turning point from unloading to loading, i.e. at time points *T*=2, 4, 6 and 8 s (Fig. 2c).

### 3.2 Large spherical indenter: *W*^*F*^/*W*^*T*^

**Fig. 3:**
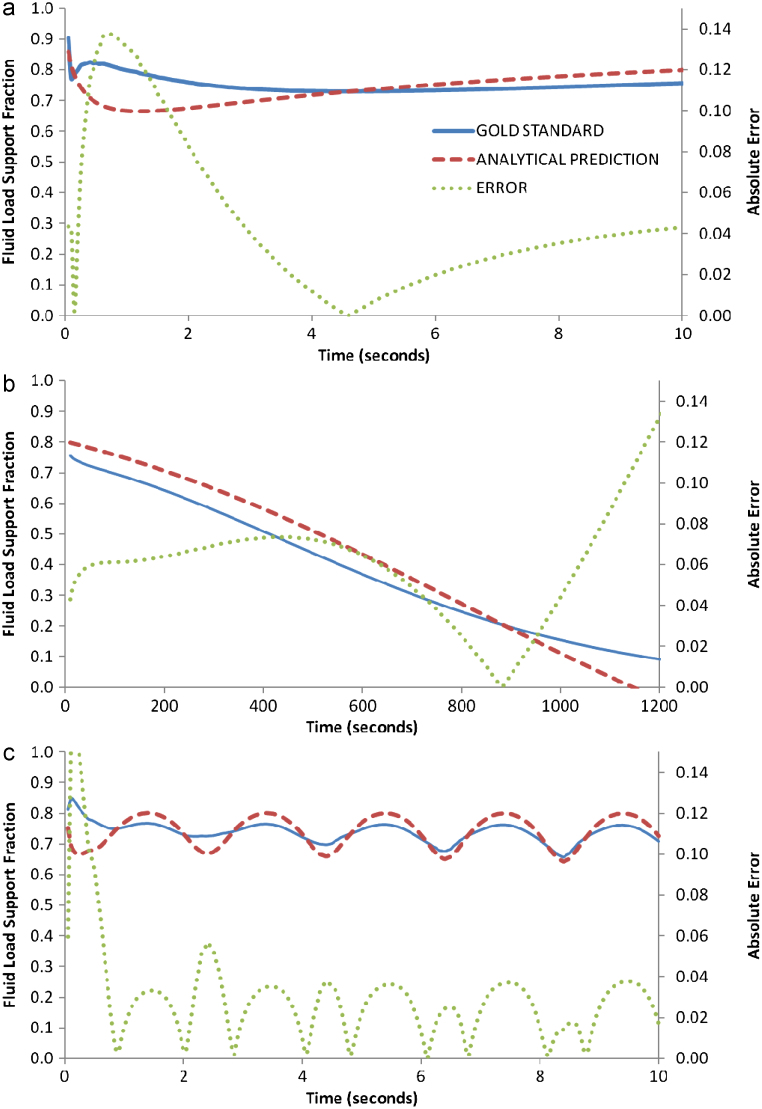
Values of the fluid load support fraction (*W*^*F*^/*W*^*T*^) calculated by a poroelastic FE simulation “gold standard” and predicted by an analytical calculation of the elastic contact mechanics (shown with errors defined as $\sqrt{{\left(y_{i}^{G}-y_{i}^{A}\right)}^{2}}$) during a series of loads with a large spherical indenter: (a) shows a linear ramp load up to 2.5% compressive strain (equivalent to 16 MPa load), (b) shows a constant 2.5% strain and, (c) shows a sinusoidal strain varying from 0.5 to 2.5% strain.

The predicted values of *W*^*F*^/*W*^*T*^ demonstrated *S*=0.05 during the ramp loading (Fig. 3a), and *S*=0.06 for the constant 2.5% strain (Fig. 3b), while the sinusoidal loading demonstrated values in line with the ‘gold standard’, but with discrepancies at the unloading-loading turning points, *S*=0.05 (Fig. 3c).

### 3.3 Small spherical indenter: *W*^*F*^/*W*^*T*^

**Fig. 4:**
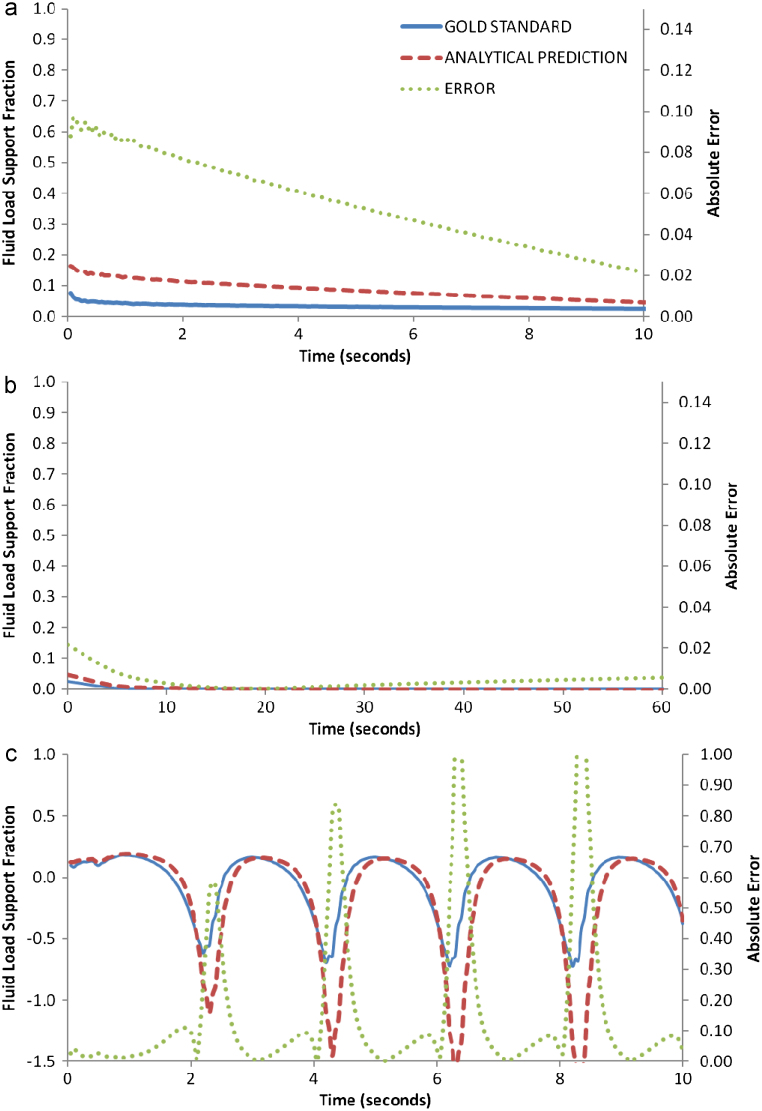
Values of the fluid load support fraction (*W*^*F*^/*W*^*T*^) calculated by a poroelastic FE simulation “gold standard” and predicted by an analytical calculation of the elastic contact mechanics (shown with errors defined as$\sqrt{{\left(y_{i}^{G}-y_{i}^{A}\right)}^{2}}$) during a series of loads with a small spherical indenter: (a) shows a linear ramp load up to 2.5% compressive strain (equivalent to 0.02 MPa load), (b) shows a constant 2.5% strain and, (c) shows a sinusoidal strain varying from 0.5 to 2.5% strain. Note, the negative *W*^*F*^/*W*^*T*^ values shown in Fig. 4c, are a result of suction at the contact surface, caused by negative pore pressures during cyclic loading [Krishnan et al., 2005].

The predicted values of *W*^*F*^/*W*^*T*^ produced *S*=0.05 during the ramp loading (Fig. 4a), and *S*=0.01 for the 2.5% strain (Fig. 4b). Again, the *W*^*F*^/*W*^*T*^ for the sinusoidal strain proved similar to the ‘gold standard’, yet with discrepancies at the unloading-loading time points, *S*=0.16 (Fig. 4c).

## 4 Discussion

The analytical prediction of *W*^*F*^/*W*^*T*^ proved, in general, accurate. The planar-surface configuration demonstrated the most desirable results (Fig. 2), while the spherical indenters also proved acceptable (Figs. 3 and 4). However, important discrepancies occurred for the sinusoidal loading configuration for all indenters, and in particular, at the instances when unloading turned to loading (*t*=2, 4, 6 and 8 s in Figs. 2c, 3c and 4c). It is interesting to note, that this analytical prediction was also capable of capturing the negative *W*^*F*^/*W*^*T*^ values during cyclic loading (Fig. 4c), which as Krishnan et al. (2005) has suggested, is an intriguing and counter-intuitive response by which cartilage supports load.

### 4.1 Limitations

Interestingly, the level of accuracy provided by the analytical approach varied for the three indenter types. This variation most probably arose from the accuracy (or more precisely, the correct use of) the elastic contact equations. For example, eq. (4) assumes a set of boundary conditions that require a rigid sphere loaded on a thin layered material that is bonded to a rigid foundation, where 0≤a/t≤20and 0≤*v*_∞_≤0.5, [Jaffar (1997)]. Given that the large spherical indenter exhibited a range of *a*/*h* ratios during the ramp loading, from ∼0.001 at the instant of load application to a maximum of 6.32 at 2.5% strain, the relatively high absolute errors (*S*=0.14) in Fig. 3a may arise from a non-conformance of the boundary conditions required by Eq. (4), i.e. at the instant of load application *a*/*h*→0 and a conflict between the boundary conditions and the mechanics arose. This predicament is likely to occur in many scenarios when, for example, the bearing surfaces do not fully conform to either the idealised dimensions or the perfect contact, defined in the given contact equation. Thus, it is prudent to note the importance of implementing the correct elastic contact model in order for this analytical *W*^*F*^/*W*^*T*^ calculation to be effective, and consequently, it could be beneficial to derive the elastic contact mechanics from configuration-specific force-deformation data. For scenarios that involve complex geometries, such as a human joint, an elastic FE simulation could provide such information.

A limitation of the viscoelastic simulations was the need to determine the time-constant parameter for each specific geometry (Table 1); as the contact area varied for each indenter geometry, the boundary flow conditions also varied (the contact interface had effectively a no-flow condition due to the impermeable representation of the indenter), and thus, the cartilage exhibited different rates of interstitial fluid pressure reduction for each indenter geometry. However, the instantaneous and the equilibrium moduli were both consistent across all indenters. Thus, when applying this technique to an in-vivo setting, the time constant is the only parameter that may prove difficult to ascertain; the moduli can be readily obtained from experimental studies or published sources. Given the complex boundary conditions present in-vivo (the biphasic jump condition), it may be worthwhile undertaking a computational poroelastic study to acquire this data (an example can be found in [Pawaskar et al., 2010)]. However, we believe this inconvenience is overridden by the benefit of being able to calculate *W*^*F*^/*W*^*T*^ in a dynamic setting.

### 4.2 Applications

The analytical *W*^*F*^/*W*^*T*^ prediction can be employed in many scenarios where the total load *W*^*T*^ and the penetration depth *δ* are known. Here, a viscoelastic FE simulation was employed to offer an example, and intended, application. However, an experiment that measures *W*^*T*^ and *δ* can apply this technique, and likewise, an explicit FE simulation that uses time-dependent mechanics can also employ such a method. What is important, is that the proposed technique measures/calculates *W*^*T*^ and *δ*: future calculations of *W*^*F*^/*W*^*T*^ do not need to arise solely from counter-sunk pressure sensors in expensive experiments, or from time-consuming poroelastic FE simulations.

The proposed analytical method is intended for the use in explicit FE and multibody simulations. It is hoped that this technique will provide essential information on material mechanics within a whole joint during physiological loading conditions. The ability to understand *W*^*F*^/*W*^*T*^ in a dynamic loading environment is imperative to understand how degenerative conditions progress and for the design of replacement therapies (medical implants).

## 5 Conclusions

The analytical technique provided a suitable means for first approximation of *W*^*F*^/*W*^*T*^, and in situations where micromechanical continuum poroelastic models cannot be employed, this technique offers a calculation with a reasonable level of confidence. This verified technique is not intended to replace current experimental or poroelastic FE modelling techniques, but rather to offer an alternative approach for when these traditional calculation techniques are impractical.

## Conflict of interest statement

There are no conflicts of interest.

## Figures and Tables

**Table 1:** Material Properties and Loading Configurations.

Material formulation	Equilibrium elastic modulus *E*_∞_ (MPa)	Poisson's ratio *ν*_∞_	Permeability (m^4^/N s)	Fluid content (%)
Poroelastic	4	0.15	1x10^−15^	75

Material formulation	Instantaneous elastic modulus *E*_0_ (MPa)	Equilibrium elastic modulus *E*_∞_ (MPa)	Time decay τ (sec)	Poisson's ratio *ν*_0_

Viscoelastic	5.19	4	*Planar* 677	0.49
*Large sphere* 469
*Small sphere* 14

	Planar surface (Ø=20 mm)	Large spherical (Ø=400 mm)	Small spherical (Ø=0.2 mm)
Loading configurations			
Ramp (strain/sec) [*equivalent pressure*]	0.0025[3.2*5 *MPa/s]	0.0025[1.65 MPa/s]	0.0025[0.002 MPa/s]
Constant strain	0.025	0.025	0.025
Sinusoidal	c_1_=0.01	c_1_=0.01	c_1_=0.01
(c_1_sin(*πt*)+c_2_)	c_2_=0.015	c_2_=0.015	c_2_=0.015

Mesh configurations			
Element length (mm)	2.00×10^−2^	2.00×10^−2^	7.45×10^−4^

Viscoelastic calibration error (viscoelastic force response compared to poroelastic force response)			
Mean error *S* (N)	3.37	0.37	4.21

^⁎^ The viscoelastic properties were implemented in a generalised form, where the generalised relaxation modulus was defined as $E(t)=E_{0}(1-E^{p}(1-e^{-t/\tau }))$, where *t* is time and *E*^*p*^ is the ratio of the equilibrium and the instantaneous modulus, respectively. Note the three loading configurations resulted in viscoelastic parameters with equivalent elastic properties, but with varying time decay constants. These parameters are a direct result of the contact-dependent fluid flow boundary at the contact interface: the planar surface prohibited flow over the entire upper surface, whereas the small spherical indenter prohibited flow for less than 1% of the upper surface, thus producing very different boundary conditions between each loading configuration.
